# Unmasking *Mycobacterium avium*: two case reports of cutaneous lesions in HIV patients after initiation of an integrase inhibitor-based regimen

**DOI:** 10.1186/s12879-026-12588-0

**Published:** 2026-01-21

**Authors:** Luis Camero, Lily M. Soto, David M. Flora-Noda, Lorena L. Becerra-Vivas, Adriana Guerra-Martínez, Mercedes España, Franklin E. Claro, Jacobus H. de Waard, Douglas Silva, Fhabián S. Carrión-Nessi, David A. Forero-Peña

**Affiliations:** 1 Department of Infectious Diseases, Hospital Universitario de Caracas, Caracas, Venezuela; 2 Department of Dermatology, Hospital Militar Universitario “Carlos Arvelo”, Caracas, Venezuela; 3 Department of Infectious Diseases, Hospital General “Dr. José Ignacio Baldó”, Caracas, Venezuela; 4 Department of Tuberculosis, Servicio Autónomo Instituto de Biomedicina “Dr. Jacinto Convit”, Universidad Central de Venezuela, Caracas, Venezuela; 5 Biomedical Research and Therapeutic Vaccines Institute, Ciudad Bolívar, Venezuela; 6 “Luis Razetti” School of Medicine, Universidad Central de Venezuela, Caracas, Venezuela

**Keywords:** *Mycobacterium avium* complex, Soft tissue infections, Nodules, HIV, Venezuela, Case reports

## Abstract

**Background:**

*Mycobacterium avium* complex (MAC) is the most common non-tuberculous mycobacteria (NTM) causing disease in immunosuppressed patients. People living with human immunodeficiency virus (PLHIV) typically present with disseminated infections characterized by non-specific constitutional symptoms such as fever, night sweats, and weight loss. Cutaneous lesions in disseminated MAC are rare and include panniculitis, granulomas, pustules, papules, nodules, abscesses and ulcerations, and ulcers. Here, we present two HIV patients who developed a disseminated MAC infection with skin lesions.

**Case presentation:**

A 40-year-old woman with a 10-year history of untreated HIV infection and a 47-year-old woman recently diagnosed with HIV both presented with constitutional symptoms. After routine screening for opportunistic infections and exclusion of active infection, they were started on antiretroviral therapy (ART) with tenofovir, lamivudine, and dolutegravir. One month later, both presented inflammatory skin lesions that evolved into ulcers. Case 1 presented with painful nodules of variable size on the upper and lower extremities that subsequently suppurated and ulcerated. Case 2 presented with erythematous-violaceous nodules on the trunk and limbs that progressed to ulceration with irregular borders, well defined, infiltrated, with necrotic background and fine perilesional desquamation. The secretion of the lesions was analyzed in both cases. Ziehl-Neelsen stain showed the presence of acid-fast bacilli only in case 1, while the GeneXpert MTB/RIF assay was negative in both cases. The biopsy of case 2 documented panniculitis. Non-pigmented colony growth was observed after 42 and 38 days of incubation, respectively. PCR restriction enzyme pattern analysis demonstrated the presence of MAC in both cases. Clarithromycin and ethambutol were started in both cases, and clofazimine was added in case 1, resulting in a satisfactory clinical course in both patients, as evidenced by the healing of the ulcers.

**Conclusions:**

These cases of disseminated MAC infection highlight that unmasking immune reconstitution inflammatory syndrome can present with severe cutaneous lesions shortly after the initiation of modern ART. Our report underscores the need for a high index of suspicion for NTM infections in patients with advanced HIV who develop new inflammatory syndromes, particularly in resource-limited settings where diagnostic delays for mycobacterial diseases are common.

## Background

Slow-growing species of the *Mycobacterium avium* complex (MAC) are the most common non-tuberculous mycobacteria (NTM) causing disease in immunosuppressed patients [1]. MAC was originally thought to consist of only two species: *M. avium* and *M. intracellulare*. However, advances in molecular identification have led to the description of additional species within MAC, including *M. chimaera*, *M. arosiense*, and *M. vulneris*, among others [2]. Despite these advances, most commercial laboratories do not identify specific species within the complex.

In immunocompetent individuals, MAC infections typically manifest as localized pulmonary infections [3]. In contrast, people living with human immunodeficiency virus (PLHIV) usually present with disseminated infections characterized by non-specific symptoms, such as fever and sweating [4]. The introduction of effective antiretroviral therapy (ART) has significantly reduced the incidence of MAC infections in PLHIV [5, 6]. However, localized lymphadenitis remains common in PLHIV and may result from immune reconstitution inflammatory syndrome (IRIS) [7, 8].

The presentation of cutaneous MAC infection in localized and disseminated disease may vary widely from patient to patient. Folliculitis, nodules, pustules, papules, erythematous lesions, ulcerations, panniculitis, and abscesses have been documented in immunosuppressed non-HIV patients [9, 10] and HIV patients [11, 12]. However, exclusive cutaneous or soft tissue involvement is rare. In Venezuela, studies on MAC are limited. Here, we present two cases with acquired immunodeficiency syndrome who developed a disseminated MAC infection with cutaneous lesions after initiation of ART.

## Case 1

A 40-year-old female patient was diagnosed with HIV infection in 2013 and had not received any medical follow-up or ART by her own choice since diagnosis. In September 2023, she presented with headache, vomiting, and temporal and spatial disorientation. On admission, the patient was cachectic, tachypneic, tachycardic, and dehydrated, with no palpable cervical or axillary lymphadenopathy, a normal lung examination, and no hepatosplenomegaly. She was disoriented in time and space, paraprosexic, with cognitive and behavioral changes, without signs of meningeal irritation, and with preserved muscle strength.

A complete blood count revealed normocytic hypochromic anemia and thrombocytopenia. A cerebrospinal fluid examination, including cytochemistry, cytomorphology, and adenosine deaminase, was normal. Cultures and stains for fungi, mycobacteria, viral tests (cytomegalovirus, Epstein-Barr virus, varicella-zoster virus, herpes simplex virus types 1 and 2, herpes virus type 6, and enterovirus), and cryptococcal antigen were negative. The CD4^+^ count was 5 cells/mm³ and the HIV viral load was 1,000,000 copies. The purified protein derivative assay was 0 mm. A computed tomography scan of the chest showed no pathological findings. A contrast-enhanced magnetic resonance imaging of the brain showed symmetrical white matter changes with loss of the frontal plates adjacent to the ventricles, consistent with HIV-associated leukoencephalopathy, later confirmed by the presence of John Cunningham virus in the cerebrospinal fluid. In November 2023, treatment with tenofovir, lamivudine, and dolutegravir was initiated, resulting in adequate tolerability and clinical improvement, leading to discharge.

**Fig. 1:**
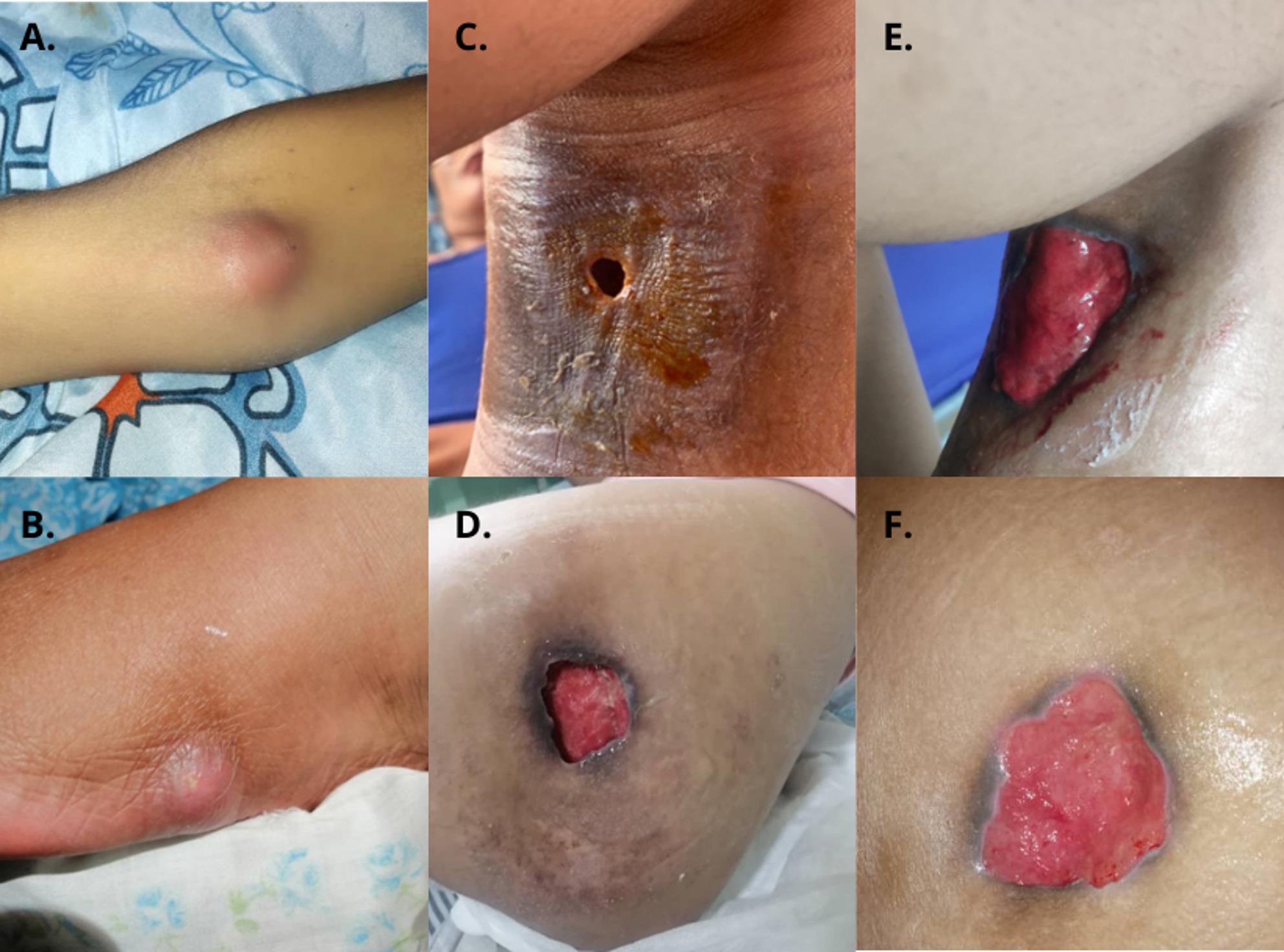
Cutaneous manifestations and evolution of case 1. (**A**,** B**) Initial nodules on the elbow and forefoot one month after starting ART. (**C**,** D**) Ulcers in the popliteal and hip regions following drainage, documented at 1 and 2 months of treatment, respectively. (**E**,** F**) Healed ulcers in the popliteal and hip regions after 6 months of treatment, demonstrating a satisfactory clinical course

One month after starting ART, the patient presented with intermittent night fever of 39 °C and multiple painful nodules that suppurated and ulcerated of various sizes in the facial region, upper extremities (elbow joint) (Fig. 1A), and lower extremities (left forefoot) (Fig. 1B), the largest measuring 6 cm in diameter. Antibiotic therapy was initiated, first with ciprofloxacin and then with vancomycin, without improvement. A specimen was obtained by aspiration of an ulnar nodule for staining and culture for fungi, aerobes, mycobacteria, and GeneXpert^®^ MTB-RIF. Ziehl-Neelsen staining revealed acid-fast bacilli, leading to initiation of anti-tuberculosis therapy with rifampin, isoniazid, ethambutol, and pyrazinamide. GeneXpert^®^ was negative. New nodules lesions appeared in the gluteal, hip, and popliteal regions. Non-pigmented colony growth was reported after 42 days of incubation at 37 °C. Polymerase chain reaction-restriction enzyme pattern analysis identified MAC.

The nodules were drained, resulting in the appearance of ulcers involving the subcutaneous tissue in the left popliteal region (Fig. 1C) and left hip region (Fig. 1D). Treatment with clarithromycin (500 mg twice daily), ethambutol (15 mg/kg daily), and clofazimine (50 mg daily) was initiated and continued for 9 months, resulting in a satisfactory clinical course as evidenced by resolution of fever and healing of the ulcers without purulent exudate (Fig. 1E and F).

## Case 2

A 47-year-old female patient presented with a one-year history of progressive and involuntary weight loss (approximately 15 kg) and diarrhea of 4 months’ duration. A diagnosis of HIV infection was made on April 13, 2024. Outpatient coproanalysis studies show *Cryptosporidium* oocysts, so she received nitazoxanide for 3 days with a resolution of the diarrhea. Initial physical examination revealed no cardiopulmonary abnormalities, adenopathy, or skin lesions. Complete blood count, renal function, and liver function were within normal limits. Due to the patient’s limited financial resources at the time of diagnosis, baseline HIV viral load and CD4^+^ cell count measurements could not be performed prior to ART initiation.

**Fig. 2:**
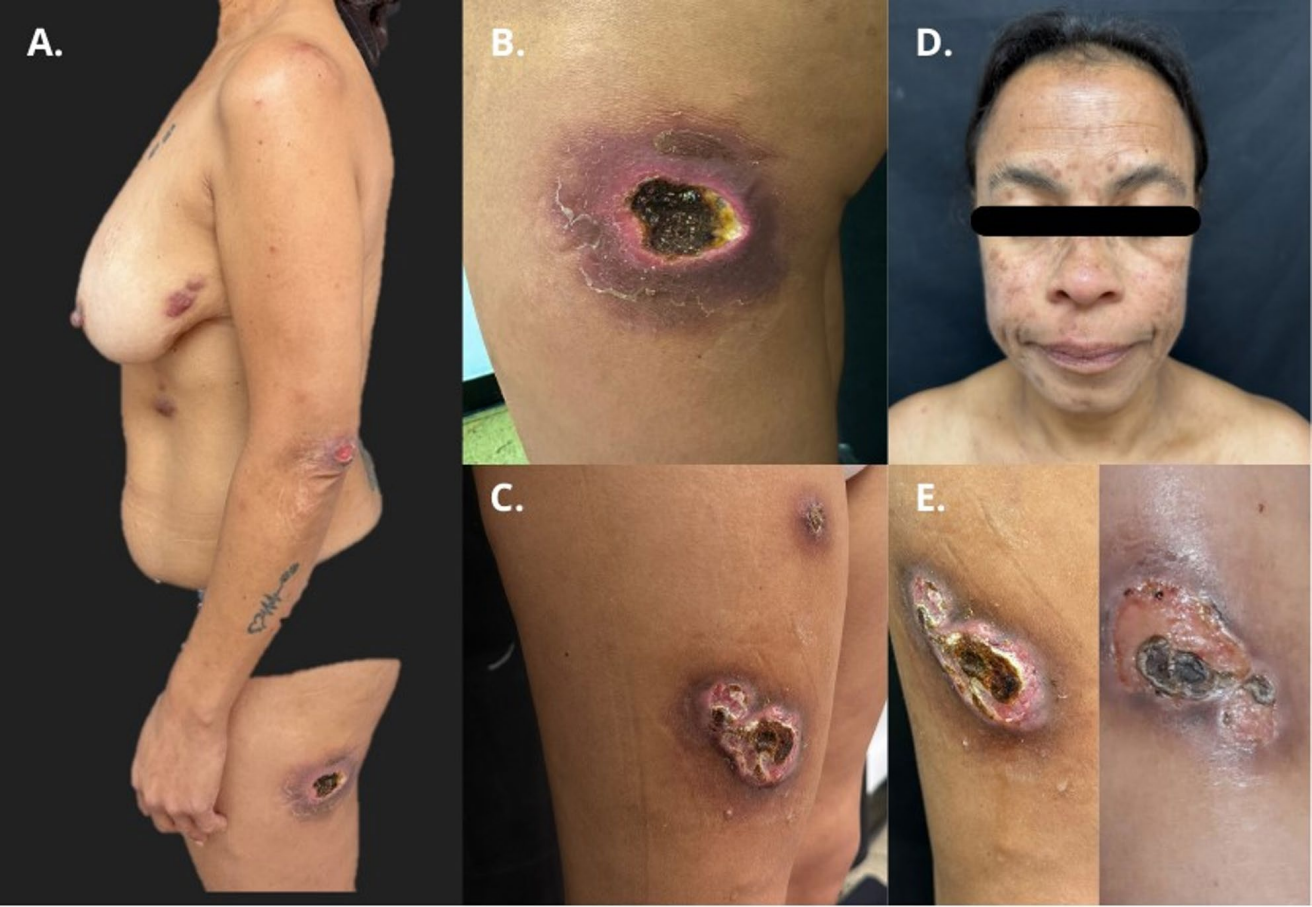
Cutaneous manifestations and course of case 2. (**A-D**) Lesions appearing one month after starting ART, including erythematous-violaceous nodules, plaques and ulcers on the trunk and lower extremities, and brown macules on the face. (**E**) Significant resolution of the lesions after one month of specific anti-MAC treatment

Treatment with tenofovir, lamivudine, and dolutegravir was initiated on April 15, 2024. One month after starting ART, the patient presented with erythematous nodules that later developed into ulcers on the lower extremities, chest, abdomen (Fig. 2A-C) and brown macules on the central facial region (Fig. 2D). The ulcers developed with irregular, well-defined, infiltrated borders with necrotic background and fine perilesional desquamation, located in the external region of the middle third of the right leg and on the lateral surface of the proximal third of the left leg. She also had erythematous-violaceous plaques with a crusty surface on the anterior surface of the right leg, hypochondrium, elbow, and left submammary region. No fever, no palpable cervical or axillary lymphadenopathy, normal lung examination, and no hepatosplenomegaly. Hematology, renal function, and liver function were within normal limits. Empirical treatment was started with clarithromycin and linezolid with partial improvement.

**Fig. 3:**
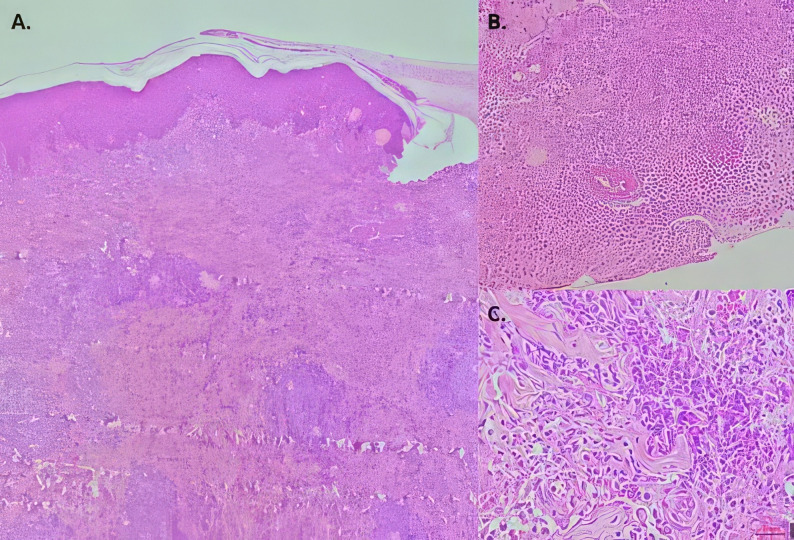
Biopsy of a lesion on the right thigh (hematoxylin and eosin staining). (**A**) Nodules of inflammatory cells distributed in the deep dermis and subcutis (original magnification × 4). (**B**) Deep portions of the fragment showing marked septal fibrosis with predominantly neutrophilic inflammatory nodules (original magnification × 40). (**C**) Predominantly neutrophilic inflammatory infiltrate with occasional lymphocytes and histiocytes (HE × 40)

She was seen by a dermatologist who decided to perform a biopsy of the right lower extremity nodule on June 4, 2024. The biopsy revealed a septal and lobular panniculitis composed of lymphocytes and neutrophils with numerous histiocytes (Fig. 3). Ziehl-Neelsen staining of ulcer secretion was negative. Non-pigmented colony growth was reported after 38 days of incubation at 37 °C. Polymerase chain reaction-restriction enzyme pattern analysis identified MAC. Treatment with clarithromycin (500 mg twice daily) and ethambutol (15 mg/kg daily) was initiated. One month after the start of treatment, the lesions resolved satisfactorily (Fig. 2E). The CD4^+^ count was 1,252 cells/mm³ (June 4, 2024) and the HIV viral load was 45 copies (July 23, 2024).

## Discussion and conclusions

Widespread use of ART has significantly reduced the incidence of MAC infection in PLHIV [5, 6]. However, a CD4^+^ count below 50 cells/µL and an HIV viral load above 1,000 copies/mL are risk factors for MAC infection in HIV patients [13]. Our two patients presented with constitutional symptoms, but only one had a very low CD4^+^ count and a high viral load. Disseminated presentation is the most common in PLHIV [4], and reports of skin and soft tissue infections are rare. Although subcutaneous nodules and abscesses due to MAC have been described in PLHIV [11, 14–17], these reports often lack detailed clinical presentation, imaging, and evolution. We present two cases of cutaneous lesions with nodules that suppurated andevolved into ulcers in different parts of the body, in contrast to the sites described in cases of MAC lymphadenitis. Histopathology of MAC skin infections shows a wide spectrum of patterns, including diffuse histiocytic infiltrate, panniculitis, nonspecific inflammatory infiltrate, and granulomas [12, 18, 19]. In case 2, our findings were compatible with panniculitis.

The diagnosis of IRIS requires the worsening of a recognized infection (“paradoxical” IR) or the emergence of a previously unrecognized infection (“unmasking” IR) in the setting of improved immune function and/or viral control [20]. The likelihood and severity of IRIS correlate with two interrelated factors: the degree of CD4^+^ cell immune suppression prior to ART initiation, and the degree of viral suppression and immune recovery following ART initiation [21–23]. Several case reports have discussed the development of MAC and other mycobacterial infections in patients receiving ART [11, 24, 25], reporting lymphadenitis within two months of treatment initiation. Cutaneous manifestations of MAC have even been identified as a sign of IRIS [11, 16, 17]. In our two cases, skin lesions started 4 weeks after ART initiation. In case 1, despite the lack of control of lymphocyte subpopulations, the initial severe immunosuppression (CD4^+^ count of 5 cells/mm³) and the recent initiation of ART suggest a clinical presentation related to IRIS. The robust immune recovery observed in case 2, with a CD4^+^ count reaching 1,252 cells/mm^3^ just two months after starting ART, may seem paradoxical for a MAC diagnosis. Classically, disseminated MAC is an opportunistic infection of profound immunosuppression, almost exclusively seen in patients with CD4^+^ counts below $50 cells/mm^3^. Our case challenges this classic paradigm and provides a critical learning point for clinicians in the current era of potent ART. The exceptionally high post-treatment CD4^+^ count is not evidence against a MAC diagnosis but is rather a hallmark of the pathophysiology of unmasking IRIS. It illustrates that clinicians cannot be falsely reassured by a normal or high CD4^+^ count in a patient who develops new inflammatory symptoms after ART initiation, as this ‘good number’ may in fact be driving the pathology. The subclinical MAC infection was likely present at baseline when the patient was severely immunocompromised. The initiation of a potent integrase inhibitor-based regimen, containing dolutegravir, led to rapid virologic suppression and a swift, powerful reconstitution of the immune system [26]. This rapid recovery “unmasked” the pre-existing pathogen, triggering an exuberant inflammatory response that manifested as cutaneous lesions [27]. This clinical sequence aligns with a compelling pathophysiological hypothesis: INSTIs achieve faster, and more profound viral suppression compared to older antiretroviral classes, and this rapid restoration of pathogen-specific immunity is a primary driver of IRIS. Several observational studies have supported this hypothesis, suggesting a higher incidence of IRIS in patients initiating INSTI-based regimens [28–30]. However, the relationship is complex, and this association has not been consistently demonstrated in all settings. Notably, a recent large-scale meta-analysis of 14 randomized controlled trials found no statistically significant difference in the overall risk of IRIS between patients starting INSTI versus non-INSTI regimens [31]. This discrepancy highlights an ongoing scientific debate and underscores the value of detailed clinical reports. While population-level data from controlled trials may not show an increased risk, our cases illustrate a clinically dramatic phenotype of unmasking IRIS that can occur following the initiation of these highly effective therapies. They serve as a crucial reminder that clinicians must maintain a high index of suspicion for IRIS in patients with advanced HIV who present with new inflammatory signs and symptoms shortly after starting modern ART, regardless of the specific drug class.

To formalize the diagnosis of unmasking IRIS, we assessed both cases against the widely recognized diagnostic criteria proposed by French et al. [20, 32]. Both cases met the necessary criteria for this diagnosis, as summarized in Table 1. The presentation with cutaneous nodules and panniculitis is an atypical manifestation of MAC, fulfilling a major criterion. Both patients demonstrated a clear response to ART, with case 1 presenting with a high baseline viral load and case 2 achieving virologic suppression, satisfying the second major criterion. Furthermore, both cases met supportive minor criteria, including a profound improvement in CD4^+^ count (documented in case 2 and inferred from clinical improvement in case 1) and an intense, pathogen-specific inflammatory response at the site of the lesions.

**Table 1:** **Assessment of cases using diagnostic criteria for IRIS (French et al.** [20, 32]**)**

Criterion	Type	Evidence in case 1	Evidence in case 2	Fulfilled
Atypical presentation of an opportunistic infection in a patient responding to ART	Major	Yes. Cutaneous nodules and ulcers are a rare presentation of disseminated MAC	Yes. Erythematous-violaceous nodules, ulcers and panniculitis are atypical for MAC	Yes
Decrease in plasma HIV RNA level by at least 1 log10​ copies/mL	Major	Although a follow-up viral load was not measured at the time of lesion appearance, a drop of > 1 log10 copies/mL is virtually certain given the patient was ART-*naïve* with a baseline of 1,000,000 copies/mL and demonstrated a positive clinical response to therapy.	Yes. Viral load was suppressed to 45 copies two months after starting ART	Yes
Increased blood CD4^+^ T-cell count after ART	Minor	A follow-up CD4^+^count was not available at the time of IRIS presentation, which is a limitation. However, given the baseline CD4^+^ count of 5 cells/mm^3^, the patient’s subsequent clinical improvement and survival are strongly consistent with immune reconstitution.”	Yes. CD4^+^ count increased to 1,252 cells/mm^3^ two months after starting ART	Yes
Increase in immune response specific to the relevant pathogen	Minor	Yes. Development of large, painful nodules with neutrophilic infiltrate indicates a robust inflammatory response to mycobacterial antigens	Yes. Biopsy confirmed panniculitis with a dense inflammatory infiltrate, a classic sign of a restored immune response	Yes
Spontaneous resolution of disease without specific therapy	Minor	Not applicable. Appropriate anti-MAC therapy was initiated	Not applicable. Appropriate anti-MAC therapy was initiated	N/A

ART: antiretroviral therapy, HIV: human immunodeficiency virus, RNA: ribonucleic acid, MAC: *Mycobacterium avium* complex, N/A: not applicable

Although MAC infection triples the risk of death regardless of CD4^+^ count, no cases of MAC in PLHIV have been reported in Venezuela [33]. Because clinical and microscopic findings in HIV patients may resemble tuberculosis, a high index of suspicion is needed, especially in severely immunocompromised patients who have recently started antiretroviral therapy. The negative GeneXpert^®^ MTB/RIF results in our cases, despite a positive acid-fast smear in Case 1, are an important diagnostic clue. As this nucleic acid amplification test is specifically designed for the detection of *M. tuberculosis* complex [34, 35], a negative result in a smear-positive patient should heighten clinical suspicion for an NTM infection like MAC. While culture remains the gold standard for NTM diagnosis [36], some species, such as MAC, can be slow growing, delaying diagnosis and timely treatment. Identification of NTM species by molecular techniques such as polymerase chain reaction-restriction enzyme pattern analysis is a rapid and highly reliable method and should be considered by every clinical microbiology laboratory [37]. A limitation of our report is the absence of baseline CD4^+^ count and viral load data for case 2, which precludes a precise assessment of the patient’s immune status at the time of ART initiation. This reflects the diagnostic challenges often encountered in resource-limited settings.

We present two cases of PLHIV with rare multiple skin lesions that appeared after initiation of ART. Low clinical suspicion and limited diagnostic capacity for NTM may delay timely treatment of this disease. These cases underscore the need for improved access to mycobacterial culture and molecular diagnostics in resource-limited settings, such as Venezuela, to enable timely identification of NTM species and guide appropriate therapy.

## Data Availability

All data and materials in this article are included in the manuscript.

## Abbreviations

MAC: *Mycobacterium avium* complex
NTM: Non tuberculous mycobacteria
PLHIV: People living with human immunodeficiency virus
ART: Antiretroviral therapy
IRIS: Immune reconstitution inflammatory syndrome
